# Conversion of senescent cartilage into a pro-chondrogenic microenvironment with antibody-functionalized copper sulfate nanoparticles for efficient osteoarthritis therapy

**DOI:** 10.1186/s12951-023-02036-5

**Published:** 2023-08-08

**Authors:** Xianming Wang, Yu Cai, Cuixi Wu, Jiamin Liang, Kangning Tang, Zefeng Lin, Lingling Chen, Yao Lu, Qing Wang

**Affiliations:** 1 Department of Orthopedic Surgery, The Second Affiliated Hospital, Guangzhou Medical University, Guangzhou, Guangdong China; 2 The First School of Clinical Medicine, Southern Medical University, Guangzhou, Guangdong China; 3 Department of Orthopedics, General Hospital of Southern Theater Command of PLA, Guangzhou, Guangdong China; 4 Precision Medicine in Oncology (PrMiO), Department of Pathology, Erasmus MC Cancer Institute, Erasmus MC, Rotterdam, The Netherlands; 5 Department of Joint and Orthopedics, Orthopedic Center, Zhujiang Hospital, Southern Medical University, Guangzhou, Guangdong China; 6 Department of Endocrinology, Zhujiang Hospital, Southern Medical University, Guangzhou, Guangdong China

**Keywords:** Copper sulfide nanoparticles, Osteoarthritis, Senescent chondrocytes, Cartilage repair

## Abstract

The development of osteoarthritis (OA) correlates with the expansion of senescent cells in cartilage, which contributes to an inflammatory microenvironment that accelerates matrix degradation and hampers cartilage generation. To address OA, we synthesized small copper sulfide nanoparticles functionalized with anti-beta-2-microglobulin antibodies (B2M-CuS NPs) that catalyze the formation of toxic •OH from H_2_O_2_ via peroxidase-like activity. These B2M-CuS NPs are specifically targeted to induce apoptosis in senescent chondrocytes while showing no toxicity toward normal chondrocytes. Furthermore, B2M-CuS NPs enhance the chondrogenesis of normal chondrocytes. Thus, B2M-CuS NPs can effectively treat OA by clearing senescent chondrocytes and promoting cartilage regeneration after intra-articular injection into the knee joints of surgery-induced OA mice. This study uses smart nanomaterials to treat OA with a synergistic strategy that both remodels senescent cartilage and creates a pro-chondrogenic microenvironment.

**Supplementary Information:**

The online version contains supplementary material available at 10.1186/s12951-023-02036-5.

## Introduction

Osteoarthritis (OA) is an age-related disease and the most common arthritis (affecting approximately 20% of adults worldwide), though effective therapies are lacking [1–3]. Cartilage degradation, the leading feature of OA progression, results from a homeostatic disruption in the balance between degradation and synthesis of extracellular matrix components. Recent studies of OA cartilage homeostasis have focused on the increase in senescent cells in articular cartilage [4–6] can result from mitochondrial dysfunction, excessive mechanical loading, oxidative stress, and DNA damage [7–10]. Senescent chondrocytes have a senescence-associated secretory phenotype (SASP) and release harmful pro-inflammatory cytokines (e.g., IL-1β, IL-6, and TNF-α) and proteases into the cartilage microenvironment that degrade the extracellular matrix [11–13]. Preclinical studies have found that the development of post-traumatic OA is delayed after local clearance of senescent cells in the articular cartilage of p16-M3R transgenic mice [14], and anti-senescence drugs (i.e., senolytics) also show potential in OA treatment [15–17]. For example, navitoclax reduces senescent chondrocytes by increasing caspase-3 levels in mouse-derived cartilage explants [18]. Intra-articular injection of the senolytic UBX0101 eliminates senescent cells and alleviates articular cartilage degradation in mice with post-traumatic OA [14]. However, a randomized, double-blind Phase 2 trial of a single intra-articular dose of UBX0101 in 183 patients failed to improve knee pain and function [19], and concerns have arisen regarding the specificity, safety, and tolerability of the systemic use of senolytics [20–22]. It is also unclear whether cartilage homeostasis can be recovered after the clearance of senescent cells. In this study, we develop a new strategy to enhance OA therapy by using nanoparticles (NPs) to convert the senescent microenvironment of articular cartilage (with accelerated cartilage degradation) into a pro-chondrogenic environment that favors cartilage generation.

To this end, we chose NPs based on copper (Cu). Copper is abundant and an essential trace element with many functions in humans, and Cu-based antimicrobial and antitumor NPs have been developed that convert reactive oxygen species (ROS) into toxic hydroxy radicals (•OH) [23, 24]. Notably, mitochondrial dysfunction, a hallmark of senescent chondrocytes in OA, induces intracellular ROS production that causes oxidative stress. Overproduction of hydrogen peroxide has been detected in the cartilage of OA patients [25], and enzyme-like Cu-based NPs would favor the elimination of senescent chondrocytes. Moreover, our previous studies indicate that Cu-based materials can promote mesenchymal stem cell chondrogenesis and cartilage repair [26–28]. Hence, Cu-based materials promote a chondrogenic microenvironment in articular cartilage after the clearance of senescent chondrocytes.

**Scheme 1:**
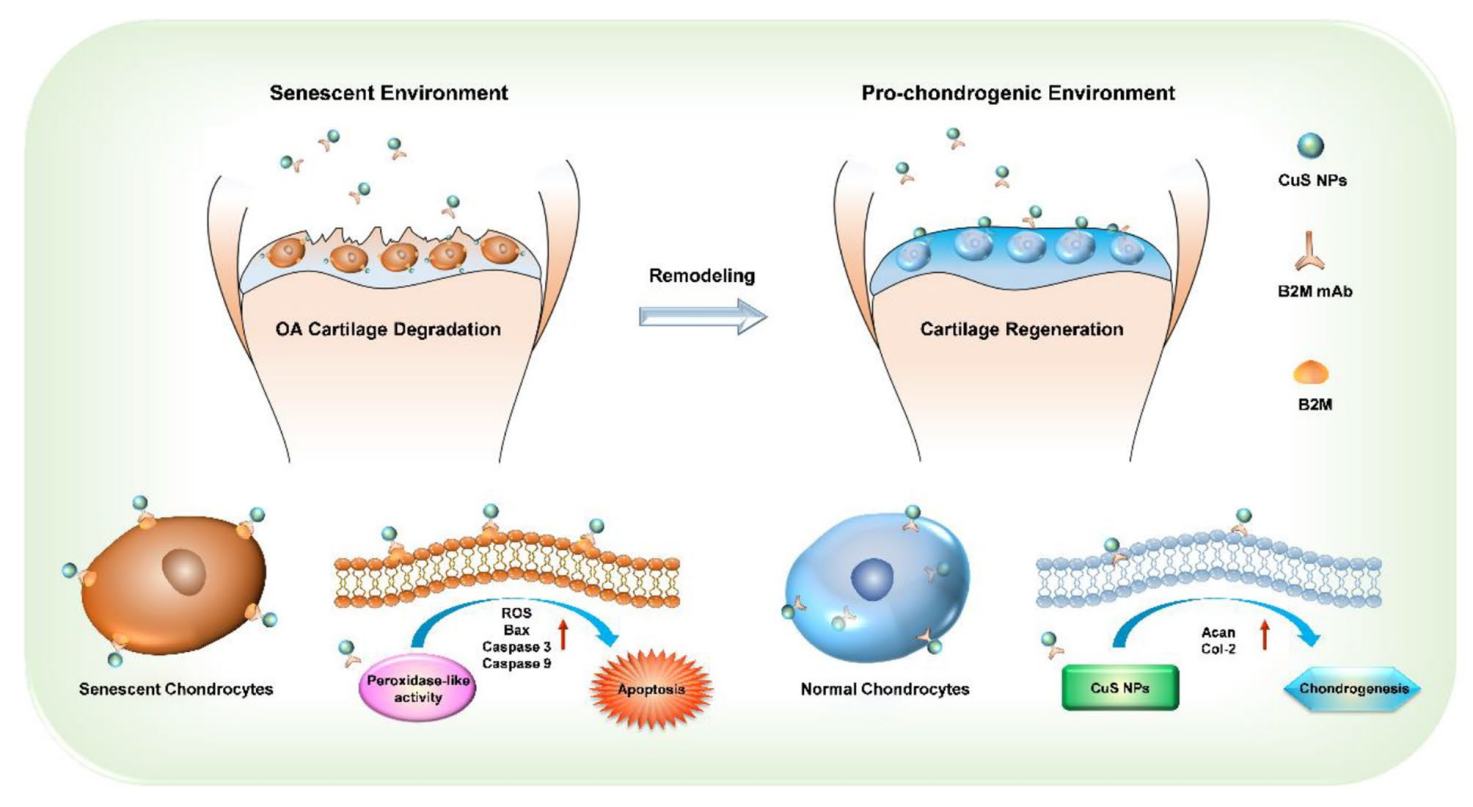
Schematic illustration of the application of B2M-CuS NPs in OA therapy. CuS NPs were functionalized with B2M antibody. The resultant B2M-CuS NPs have peroxidase-like activity and selectively target senescent chondrocytes for elimination while promoting chondrogenesis. Therefore, B2M-CuS NPs may be a low-toxicity solution for the generation of a pro-chondrogenic microenvironment that supports the remodeling and regeneration of senescent cartilage in OA.

In our approach, we first synthesized ultrasmall CuS NPs to penetrate articular cartilage, and these NPs were then conjugated with an antibody against beta-2 microglobulin (B2M) on senescent chondrocytes. The resultant B2M-CuS NPs can eliminate senescent chondrocytes via a peroxidase-like activity that converts H_2_O_2_ into toxic •OH and accelerates apoptosis. These B2M-CuS NPs are selectively targeted and therefore safe for normal chondrocytes while also facilitating chondrogenesis. After intra-articular injection into the knee joints of surgery-induced OA mice, B2M-CuS NPs successfully reduced articular cartilage erosion without causing any adverse effects. Thus, B2M-CuS NPs may be effective OA nanotherapeutics that can selectively clear senescent chondrocytes while promoting cartilage repair (Scheme 1).

## Materials and methods

### Materials

Anhydrous copper chloride (CuCl_2_), 3,3’,5,5’-tetramethylbenzidine (TMB), bovine serum albumin (BSA), and sodium sulfide nonahydrate (Na_2_S·9H_2_O) were from Macklin Biochemical (Shanghai, China). Doxorubicin (Dox), N-(3-Dimethylaminopropyl)-N’-ethylcabodiimide hydrochloride (EDC), and N-hydroxysuccinimide (NHS) were from Sigma-Aldrich (USA). Antibodies against p16^ink4a^ (polyclonal, ab108349), B2M (monoclonal, ab75853), high mobility group protein 1 (HMGB-1) (ab228624), matrix metalloprotein 13 (MMP-13) (ab39012), and type II collagen (Col-2) (ab34712) were from Abcam (UK). The cellular senescence β-galactosidase staining kit and cell counting kit (CCK-8) were from Beyotime Biotechnology (Shanghai, China). FITC-xtra and MitoROS™ 580 were from ATT Bioquest (USA). The Evo M-MLV Reverse Transcription Reagent and SYBR Green Pro Taq HS qPCR Kit were from Accurate Biology (China). The chondrogenic differentiation kit was from Cyagen Biosciences (USA).

### Synthesis of CuS NPs and B2M-CuS NPs

CuS NPs were synthesized by a previously reported method, with some modifications [29]. Briefly, 100 µL of CuCl_2_ (1 M, 13.445 mg) solution was added to 10 mL of BSA (10 g L^− 1^) solution and stirred at room temperature (RT, 25 °C) for 5 min. Then, 100 µL of Na_2_S solution (1 M, 7.804 mg) was added dropwise over 5 min into the above mixture under continuous stirring. The mixture was stirred at 90 °C until a dark green color formed, and free BSA and ions were removed by centrifugation and dialysis against ddH_2_O.

To prepare the B2M-CuS NPs, B2M antibody was conjugated to CuS NPs through an EDC/NHS reaction [30, 31] with a 1:3 antibody:NP molar ratio. Unmodified B2M antibody was removed by repeated centrifugation for 10 min at 10,000 rpm. The resultant B2M-CuS NPs were stored at -20 °C before use.

### Characterization

The morphology of CuS NPs was observed by high-resolution transmission electron microscopy (HRTEM; JEOL JEM 2100 F, Japan). The crystalline structure of CuS NPs was determined by X-ray diffraction (XRD; Rigaku Smartlab 9KW, Japan), and absorption spectra were obtained by a spectrophotometer (Thermo Scientific Multiskan GO, USA). The valence states of Cu in NPs were studied by X-ray photoelectron spectroscopy (XPS; Thermo Scientific K-Alpha). The zeta potential and hydrated particle size of CuS NPs and B2M-CuS NPs were determined using a nano-analyzer (Malvern Zetasizer Nano ZS90, UK).

### Peroxidase-like activity

The peroxidase-like activity of CuS NPs was evaluated as previously reported using TMB as a substrate [24]. For the detection of H_2_O_2_-dependent oxidation of TMB, different reagent combinations were set, including (1) TMB + H_2_O_2_, (2) TMB + NPs, and (3) TMB + H_2_O_2_ + NPs. CuS NP solutions (100 µL, 100 µg mL^− 1^) were diluted with HAc/NaAc buffer (0.2 M:0.2 M, pH = 4.6). Then, 100 µL of TMB solution and 100 µL of H_2_O_2_ (10 mM) in dimethyl sulfoxide (2 mM) were added to the NP solutions. After incubation at RT for 30 min, the absorbance of the samples was recorded (BioTek Synergy H1, USA).

**Fig. 1:**
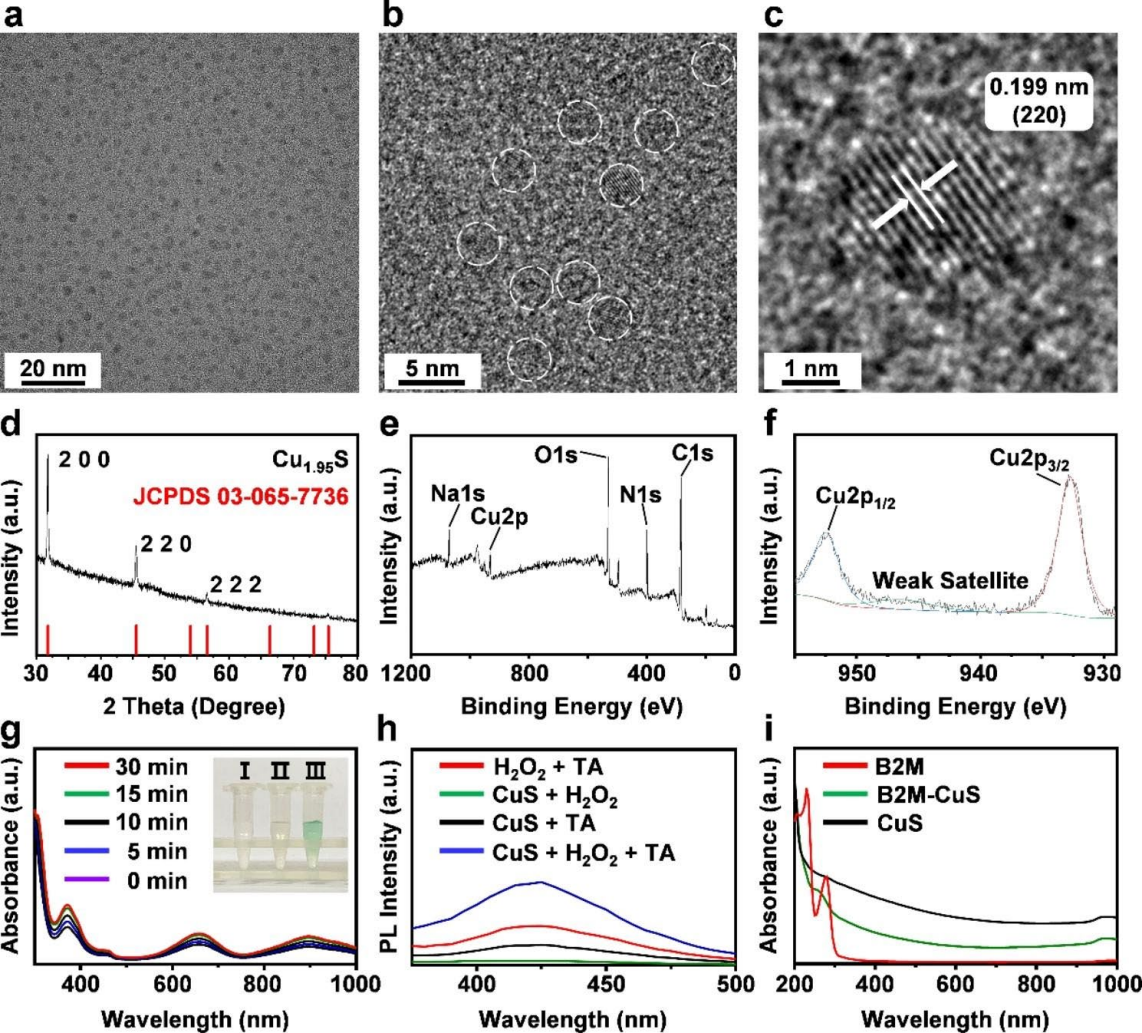
Characterization and enzyme-like activity of NPs. **(a, b)** HRTEM images of CuS. NPs are highlighted with white dotted lines. **(c)** The lattice spacing of CuS NPs. **(d)** XRD pattern of the synthesized CuS NPs corresponds with the standard Cu_1.95_ S crystal phases. **(e, f)** XPS spectra of CuS NPs. **(g)** Peroxidase-like activities of CuS NPs at different time intervals (0, 5, 10, 15, and 30 min). Inset: (I) TMB + H_2_O_2_, (II) TMB + CuS NPs, and (III) TMB + H_2_O_2_ + CuS NPs. The solution of CuS NPs + H_2_O_2_ + TMB generates blue reaction products from the oxidation of TMB, indicating the peroxidase-like activity of CuS NPs. **(h)** Fluorescence spectra after the reaction of CuS, TA, and H_2_O_2_, demonstrating that CuS NPs catalyze ∙OH formation from H_2_O_2_. **(i)** Absorption spectra of B2M antibody, CuS, and B2M-CuS NPs.

### Establishment of senescent chondrocytes

The mouse embryonic carcinoma-derived chondrogenic cell line ATDC5 was used for in vitro studies. ATDC5 cells were cultured in Dulbecco’s Modified Eagle’s Medium (DMEM; Gibco, USA) supplemented with 10% fetal bovine serum (FBS; VivaCell, Shanghai, China) and 1% penicillin/streptomycin at 37 ℃ under 5% CO_2_. Senescent chondrocytes were established by Dox treatment according to previous reports [32, 33]. Briefly, ATDC5 cells were treated twice with 0.1 µM Dox (48 h treatment interval), and senescent chondrocytes were established after 4 days of stimulation.

### Immunofluorescence staining of senescent cells

Normal and DOX-induced senescent ATDC5 cells were fixed with 4% paraformaldehyde for 15 min, permeabilized with phosphate-buffered saline (PBS) containing 0.25% Triton X-100 for 10 min, and then blocked with 4% (v/v) BSA for 30 min at RT. Cells were then incubated with primary antibody overnight at 4 ℃, washed three times with PBS, and then incubated with the corresponding secondary antibody for 2 h. After washing with PBS, cell nuclei were stained with 4,6-diamino-2-phenyl indole (DAPI) for 5 min, and then cells were imaged by fluorescence microscopy.

**Fig. 2:**
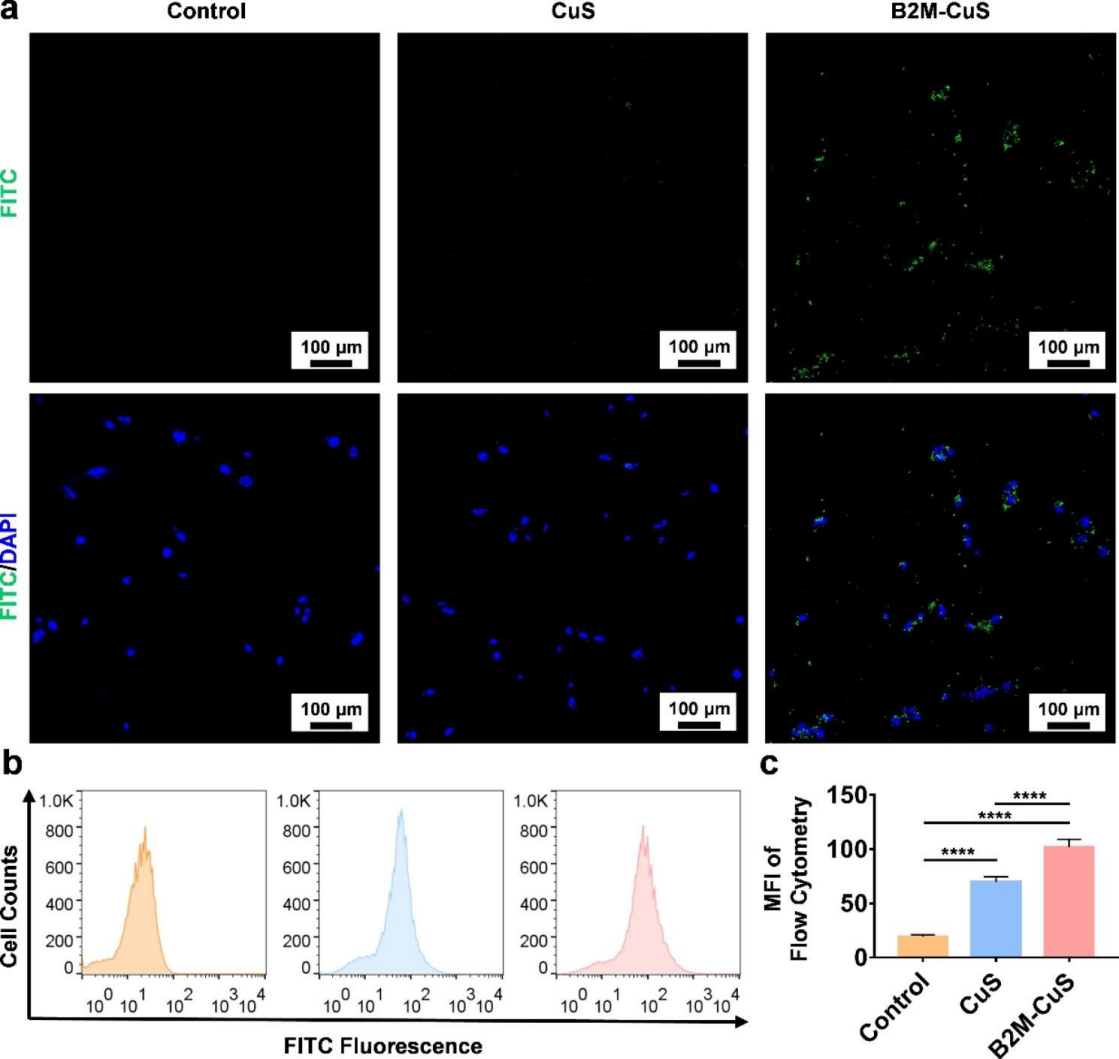
Targeting of senescent cells by B2M-CuS NPs. **(a)** Representative fluorescent images of senescent chondrocytes co-cultured with FITC-labeled CuS or B2M-CuS NPs. Stronger green fluorescence (the FITC-labeled NPs) was observed in the B2M-CuS group relative to the CuS group. Nuclei were stained with DAPI (blue fluorescence). **(b)** Representative flow cytometry images of FITC fluorescence analysis in senescent cells co-cultured with NPs. **(c)** Quantitative analysis of the mean fluorescence intensity of FITC calculated from (b). Data are presented as mean ± SD (*n* = 3). *p<0.05, **p<0.01, ***p<0.001, and ****p<0.0001

### SA-β-galactosidase staining

To measure the marker senescence-associated β-galactosidase (SA-β-gal) [34, 35], both normal and senescent ATDC5 cells were stained using a SA-β-gal staining kit (Beyotime Institute of Biotechnology, Nanjing, China) according to the manufacturer’s instructions. Cells were washed with PBS, fixed with paraformaldehyde for 15 min at RT, and then stained overnight at 37 ℃. Images were acquired using a microscope.

**Fig. 3:**
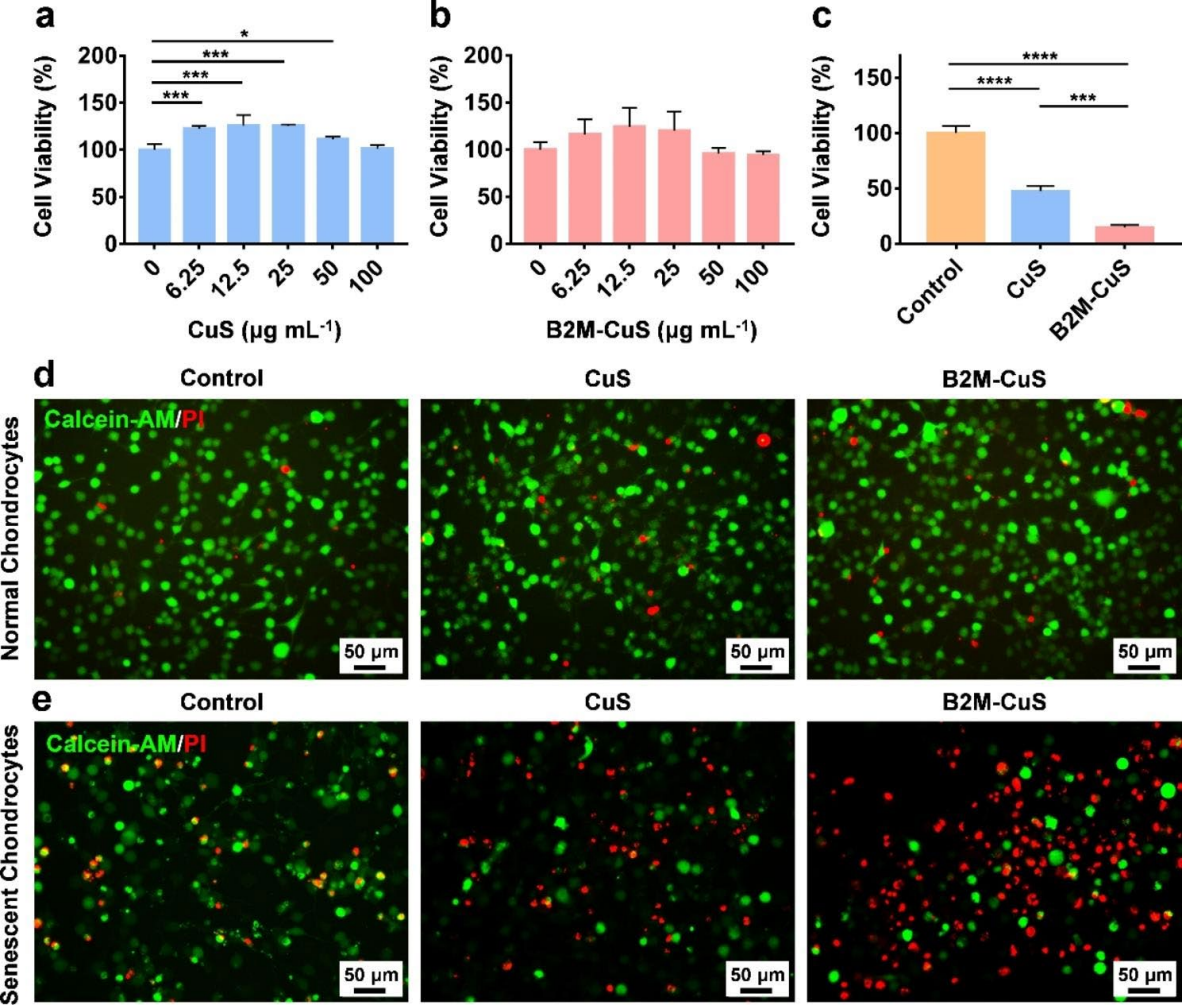
Cytotoxicity of CuS NPs toward normal and senescent chondrocytes. **(a, b)** Cell viability of normal chondrocytes co-cultured with CuS and B2M-CuS NPs at various concentrations for 24 h. **(c)** Cell viability of senescent chondrocytes co-cultured with CuS or B2M-CuS NPs (50 µg mL^− 1^) for 24 h. Live/dead staining of normal chondrocytes **(d)** and senescent chondrocytes **(e)** co-cultured with CuS NPs or B2M-CuS NPs (50 µg mL^− 1^) for 24 h. Data are presented as mean ± SD (*n* = 3). *p<0.05, **p<0.01, ***p<0.001, and ****p<0.0001

### Cytotoxicity of the NPs

The cytotoxicity of CuS and B2M-CuS NPs was investigated by the standard CCK-8 method. Normal ATDC5 cells were seeded in a 96-well plate (1 × 10^4^ cells per well) and cultured with DMEM containing 10% FBS for 24 h at 37 ℃. Cells were then cultured with CuS and B2M-CuS NPs at several concentrations (100, 50, 25, and 10 µg mL^− 1^) for 24 h. Thereafter, cells were incubated with 10% CCK-8 solution for an additional 2 h and optical density (OD) values were measured at 450 nm using a microplate reader.

### Hemocompatibility of NPs

The hemocompatibility of CuS and B2M-CuS NPs was evaluated. Red blood cells (RBCs) derived from healthy C57BL/6 mouse blood were diluted with PBS (pH 7.4, 4:5 v/v). The NPs were suspended in PBS and incubated at 37 ℃ for 30 min. After incubation, 200 µL of diluted RBCs were added to the samples and incubated at 37 ℃ for an additional 1 h. PBS and ddH_2_O were negative and positive control samples, respectively. After centrifugation at 3000 rpm for 5 min, the supernatant OD545 values were measured on a microplate reader. The hemolysis ratio was calculated by the following formula: Hemolysis ratio (%) = (OD_sample_ – OD_negative_) / (OD_positive_ – OD_negative_) × 100.

**Fig. 4:**
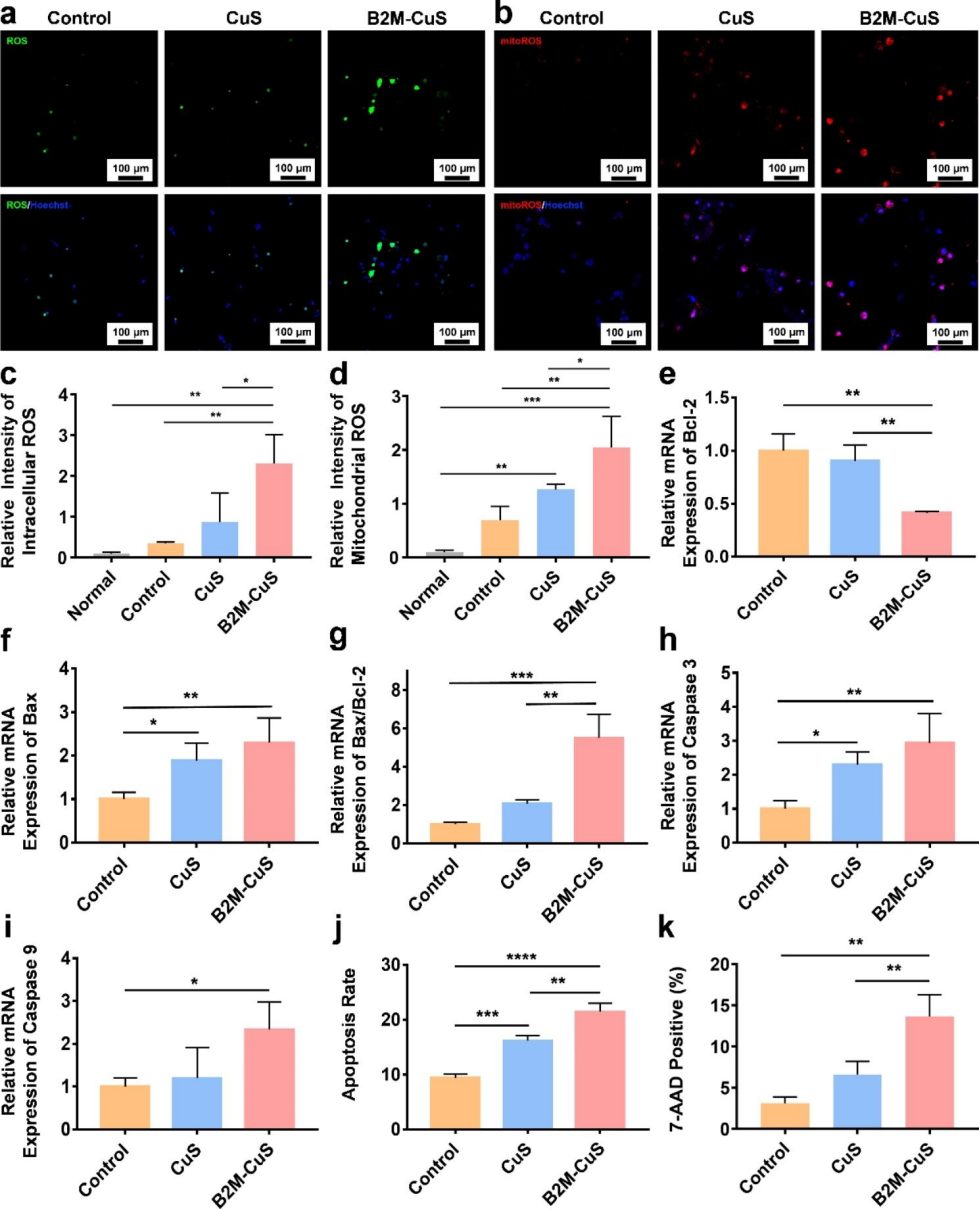
Apoptosis of senescent chondrocytes induced by B2M-CuS NPs. **(a)** Representative fluorescence images of total intracellular ROS in senescent chondrocytes after co-culturing with NPs (50 µg mL^− 1^) for 24 h. **(b)** Representative fluorescence images of mitochondrial ROS in senescent chondrocytes following treatment. **(c)** Quantitative analysis of the relative fluorescence intensity of total ROS derived from (a). **(d)** Quantitative analysis of the relative fluorescence intensity of mitochondrial ROS derived from (b). **(e-i)** Relative mRNA expression of *Bcl-2*, *Bax*, *Bax/Bcl-2*, *Caspase-3*, and *Caspase-9* in senescent chondrocytes following treatment (*n* = 3). **(j)** Quantitative analysis of apoptosis in senescent chondrocytes following treatment. **(k)** Quantitative analysis of 7-AAD positive cells in different groups. Data are presented as mean ± SD (*n* = 3). *p<0.05, **p<0.01, ***p<0.001, and ****p<0.0001

### Targeting effect of B2M-CuS NPs on senescent chondrocytes

To prepare FITC-labeled NPs, 5 µL of FITC-xtra solution (20 mM) was added to 5 mL of CuS and B2M-CuS NPs solutions (5 mg). After stirring on ice for 60 min, free FITC-xtra was removed by centrifugation for 5 min at 10,000 rpm.

To investigate the targeting specificity of B2M-CuS NPs, senescent ATDC5 cells and normal ATDC5 were seeded in a 48-well cell culture plate and grown for 24 h. Subsequently, FITC-labeled CuS and B2M-CuS NPs (50 µg mL^− 1^) were added and cultured with cells for an additional 4 h. After removing the supernatant, cells were washed with PBS, fixed with 4% paraformaldehyde for 15 min, stained with DAPI for 5 min, and visualized under a confocal laser scanning microscope (LSCM; Nikon ECLIPSE Ti2-E, Japan). As a control, senescent cells were first treated with free B2M antibody for 6 h, and then B2M-CuS NPs were added. After 24 h, cells were washed and observed. Additionally, flow cytometry was performed to detect the fluorescence intensity of treated senescent cells.

### Specific elimination of senescent chondrocytes by NPs

To study the specific elimination of senescent chondrocytes by the NPs, DOX-induced senescent ATDC5 cells were seeded on a 48-well cell culture plate (5 × 10^4^ cells per well). CuS and B2M-CuS NPs (50 µg mL^− 1^) were added to the plate and cultured with cells for 24 h, and cell viability was assayed with CCK-8. Live/dead staining was also performed. The above-treated cells were gently washed with PBS and stained with 200 µL of Calcein AM and propidium iodide (PI; BestBio, China) for 30 min at 37 ℃ in the dark. Cells were imaged with a fluorescence microscope.

### Detection of intracellular ROS and mitochondrial ROS

Senescent ATDC5 cells were cultured with CuS and B2M-CuS NPs (50 µg mL^− 1^) for 24 h, and intracellular ROS was detected via a 2′,7′-dichlorodihydrofluorescein diacetate (DCFH-DA) method as we previously reported [36, 37]. Following treatment, cells were washed with PBS and then incubated with 500 µL DCFH-DA working solution (20 µM) at 37 °C for 30 min. Nuclei were Hoechst stained for 5 min. Mitochondrial ROS was detected by MitoROS™ 580 staining according to the manufacturer’s instructions. MitoROS™ 580 working solution (200 µL) was added to each well and incubated for 10 min. Cells were washed with PBS twice and observed using LSCM. As a reference, intracellular ROS and mitochondrial ROS levels of normal chondrocytes were measured.

**Fig. 5:**
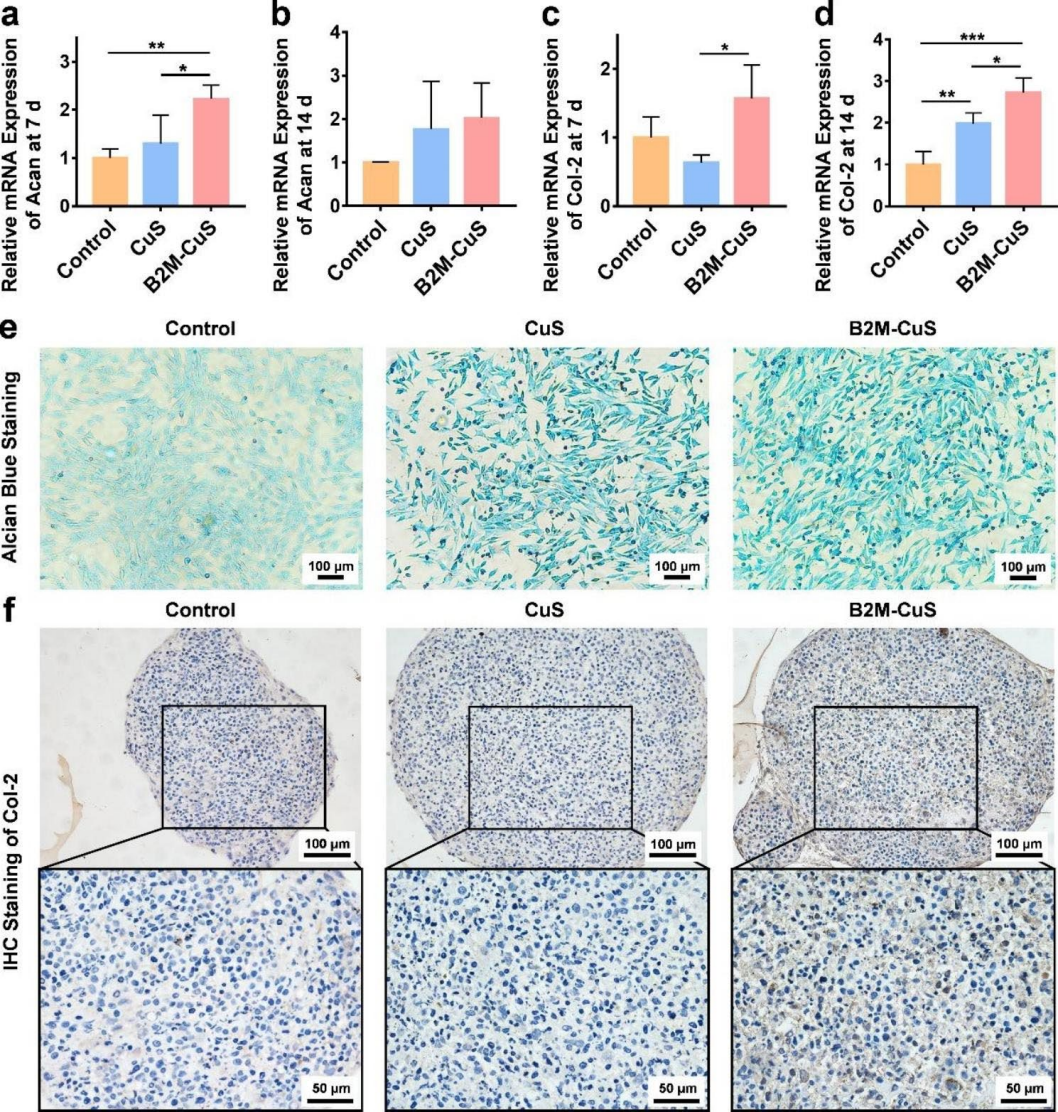
Chondro-inductivity of B2M-CuS NPs. **(a-d)** Relative mRNA expression of *Acan* and *Col-2* in chondrocytes after co-culturing with NPs for 7 and 14 days. **(e)** GAG deposition (visualized by alcian blue staining; blue color) in chondrocytes after co-culturing with NPs for 7 days. **(f)** Immunohistochemistry staining of Col-2 (brown color) in cell pellets co-cultured with NPs for 21 days. Data are presented as mean ± SD (*n* = 3). *p<0.05, **p<0.01, ***p<0.001, and ****p<0.0001

### Apoptosis and necrosis of senescent chondrocytes

After culturing with CuS and B2M-CuS NPs (50 µg mL^− 1^) for 24 h, senescent ATDC5 cells were collected and incubated with 100 µL of Annexin V-FITC and PI solution (BD, USA) for 20 min at RT. Cell apoptosis and necrosis were measured by flow cytometry and the results were analyzed with FlowJo X. To investigate the elimination of senescent cells by NPs, treated cells were stained with 7-aminoactinomycin D (7-AAD) for 30 min and then analyzed by flow cytometry.

The expression levels of apoptosis-related genes (e.g., *Bax*, *Bcl-2*, *caspase-3*, and *caspase-9*) were determined by real-time quantitative PCR (RT-qPCR). Total RNA from treated senescent ATDC5 cells was extracted with Trizol (Thermo Fisher Scientific), and RNA concentrations were determined by spectrophotometry (Nanodrop 2000; Thermo Fisher Scientific). RNA was reversed transcribed into cDNA using the Evo M-MLV reagent kit according to the manufacturer’s instructions. The cycling protocol was: 95 ℃ for 15 min, followed by 45 cycles of 95 ℃ for 5 s and 60 ℃ for 30 s. The results were analyzed with Rotor-Gene Real-Time software. Relative mRNA expression levels of each gene were normalized to glyceraldehyde-3-phosphate dehydrogenase (*GAPDH*) levels using the 2^−ΔΔCT^ method. Primers used for RT-qPCR are listed in Table S1.

### Chondrogenic inductivity of the NPs

To evaluate the in vitro chondrogenesis induced by NPs, ATDC5 cells were cultured with chondrogenic medium (MUXMX-90,041, Cyagen Bioscience, USA) containing CuS NPs or B2M-CuS NPs (50 µg mL^− 1^). After 7 days, cells were washed with PBS, fixed with 4% paraformaldehyde for 15 min, and stained with alcian blue 8GX solution for 30 min at RT to detect intracellular glycosaminoglycan (GAG). After washing with PBS three times, cells were observed with a microscope.

To determine the expression level of chondrogenesis-related genes *aggrecan* (*Acan*) and *Col-2*, cells were collected after 7 and 14 days of chondrogenic induction and RT-qPCR was performed.

In addition, ATDC5 cells were induced into cell pellets, as previously reported, and cultured in chondrogenic medium with NPs (50 µg mL^− 1^) [38]. The medium was changed every 3 days. After 21 days, cell pellets were collected and fixed in 4% paraformaldehyde, embedded in paraffin, and cut into sections. Col-2 expression was detected by immunohistochemistry.

**Fig. 6:**
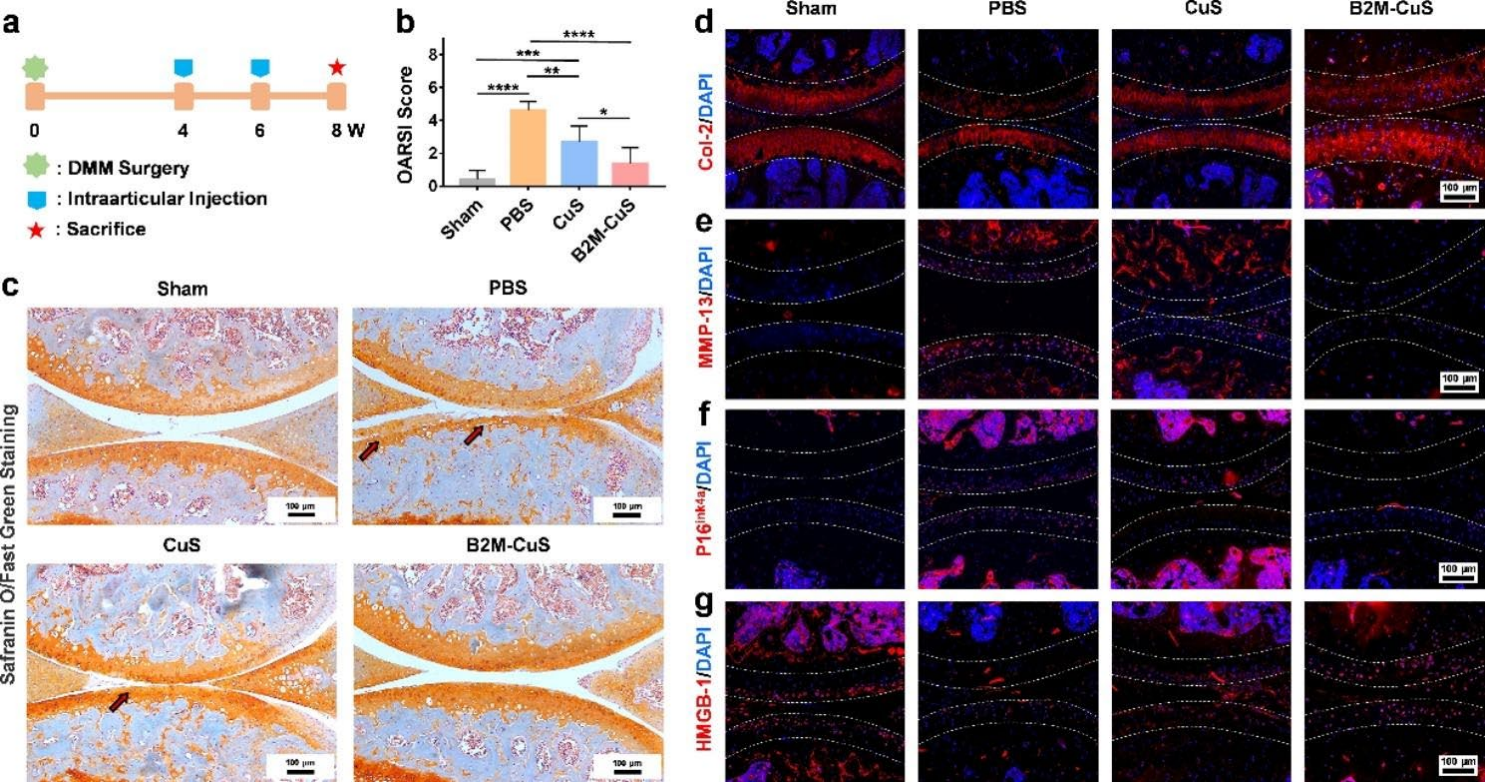
Therapeutic effects of B2M-CuS NPs in a surgery-induced OA mouse model. **(a)** Schematic diagram of the experimental design. B2M-CuS NPs, CuS NPs, and PBS were intra-articularly injected into the knee joints of OA mice every 2 weeks beginning at 4 weeks post-surgery. Knee joints were collected at 8 weeks post-surgery. **(b)** OARSI scores were measured from histological sections. **(c)** Representative images of Safranin O/fast green staining of the knee joints after treatment. Cartilage lesions are highlighted by red arrows. **(d–g)** Immunofluorescence staining of knee joints at 8 weeks post-surgery. The expression of Col-2 **(d)** was higher while the expression of MMP-13 **(e)** was lower in the B2M-CuS group relative to the CuS and PBS groups. Senescence marker p16^ink4a^**(f)** was highly expressed while HMGB-1 expression **(g)** was negligible in the PBS group. The expression of p16^ink4a^ and HMGB-1 were similar between B2M-CuS and sham groups, indicating that B2M-CuS NPs effectively eliminated senescent chondrocytes. Cartilage areas are highlighted by white dotted lines. Data are presented as mean ± SD (*n* = 6). *p<0.05, **p<0.01, ***p<0.001, and ****p<0.0001

### Therapeutic effects of the NPs in an OA mouse model

All animal studies were approved by the Institutional Animal Care and Use Committee (IACUC) of the General Hospital of Southern Theater Command of PLA (2021032401). Adult male C57BL/6 mice (20 g, 12 weeks) were anesthetized and induced into surgical destabilization of the medial meniscus (DMM) [38]. Briefly, the medial meniscus supporting ligament (MMSL) was resected after a medial capsular incision was made on the right knee joint of mice. After 4 weeks, OA mice models were established. The control group received sham surgery (opening of the joint capsule without MMSL resection; *n* = 6).

The OA mice were randomly divided into three treatment groups (*n* = 6 per group): (1) PBS, (2) CuS NPs, and (3) B2M-CuS NPs. The NPs (10 µL, 2.5 mg kg^− 1^) and PBS (10 µL) were delivered by intra-articular injection into the right knee of the OA mice at 4 weeks and 6 weeks post-surgery, respectively. Mice were sacrificed at 8 weeks post-surgery and the right knee joints were collected for further evaluation.

### Histological analysis and biodistribution

For histological analysis, the right knee joint samples were fixed in 4% paraformaldehyde overnight, decalcified in an EDTA solution, and embedded in paraffin blocks. Sections were cut into 5 μm-thick slices and stained with hematoxylin and eosin (H&E) and Safranin O/fast green. All sections were visualized with an inverted microscope (Nikon TS100, Japan). The Osteoarthritis Research Society International (OARSI) scores were evaluated according to a previous report [39]. Immunofluorescence staining for protein expression (Col-2, MMP-13, p16^ink4a^, and HMGB-1) was performed following our previously reported methods [38, 40]. Images were obtained using an inverted fluorescent microscope. The fluorescence intensity was calculated using ImageJ software.

**Fig. 7:**
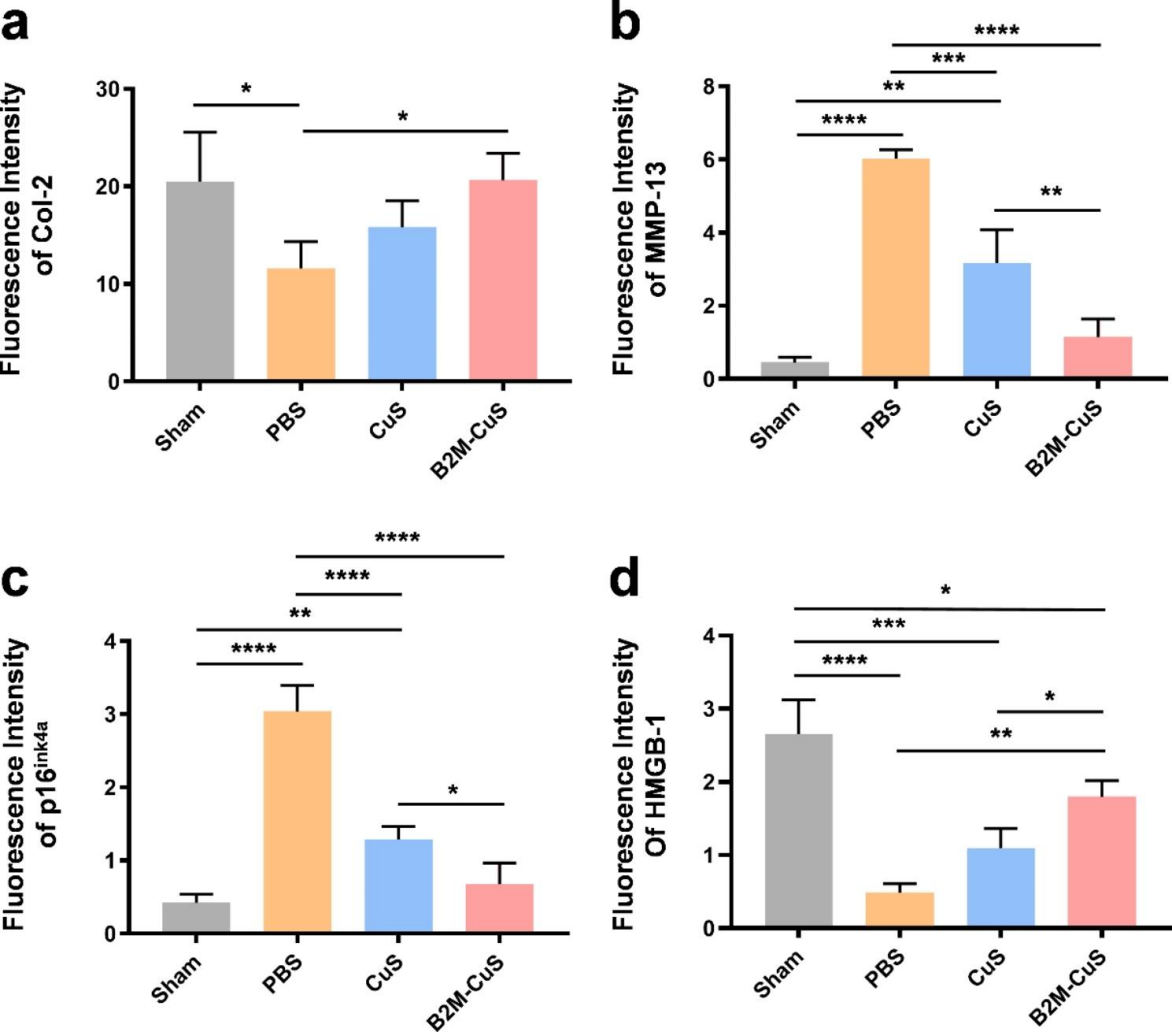
Relative fluorescence intensity calculated from the immunofluorescence-stained sections. **(a)** Col-2. **(b)** MMP-13. **(c)** p16^ink4a^. **(d)** HMGB-1. Data are presented as mean ± SD (*n* = 3). *p<0.05, **p<0.01, ***p<0.001, and ****p<0.0001

To evaluate the biodistribution of B2M-CuS NPs, mice were intra-articularly injected with the NPs. After 24 h, mice were sacrificed and the main organs (heart, liver, spleen, lung, kidney, and right knee joint) were collected and weighed. Samples were digested with aqua regia and Cu^2+^ content was determined by ICP-MS (Agilent 7700, Germany).

### Statistical analysis

Data are presented as mean ± standard deviation (SD). Statistical comparisons among different groups were performed by using one-way analysis of variance (ANOVA) followed by posthoc multiple comparisons (LSD test). A p-value < 0.05 was considered statistically significant and a p-value < 0.01 was considered highly statistically significant.

## Results and discussion

### Characterization and enzyme-like activity of CuS NPs

We first synthesized ultrasmall CuS NPs because only NPs less than 96 nm can penetrate cartilage [41]. HRTEM images (Fig. 1a and b) reveal that CuS NPs of 3 nm in size were well dispersed. The EDS spectra confirmed that the NPs are composed of Cu and S (Fig. S1). The XRD pattern (Fig. 1d) indicated that the characteristic peaks (2 0 0, 2 2 0, and 2 2 2) in the CuS NP sample are well indexed to standard Cu_1.95_ S crystal phases (JCPDS 03-065-7736). Some sample peaks are not apparent because the abundant BSA on the sample suppresses the XRD signal[42]. The lattice spacing of 1.99 Å in CuS NPs (Fig. 1c) was consistent with the d-spacing of the (2 2 0) plane of Cu_1.95_ S. XPS was also used to characterize the NPs (Fig. 1e). High-resolution spectra of Cu 2p reveal peaks at 932.53 (Cu 2p_3/2_) and 952.34 (Cu 2p_1/2_) eV, which can be assigned to CuS (Fig. 1f).

To detect the peroxidase-like activity of CuS NPs, TMB was applied as a peroxidase substrate. As shown in Fig. 1g, the TMB + H_2_O_2_ + CuS mixture turned blue after 30 min of incubation, which indicates the decomposition of H_2_O_2_ and the formation of oxidized TMB from the peroxidase-like activity of CuS NPs. Moreover, TA was used as a fluorescent indicator to measure ·OH formation. TA reacts with ·OH to form the fluorescent 2-hydroxyl terephthalate (TAOH) [43]. The fluorescence intensity (Fig. 1h) was strongest in the CuS + H_2_O_2_ + TA treatment group, indicating that ·OH was formed from H_2_O_2_ by CuS NPs. Thus, the peroxidase-like activity of CuS NPs favors ·OH formation to eliminate senescent chondrocytes.

### Senescent chondrocyte-targeting effect of B2M-CuS NPs

To enhance the targeting specificity of NPs, an antibody against B2M was conjugated with BSA-assisted CuS NPs to obtain B2M-CuS NPs. Both BSA and antibodies are rich in amino and carboxyl groups. Therefore, a classical EDC/NHS method was used to form an amide bond via condensation between BSA-CuS and the B2M antibody. Compared with uncoated CuS NPs, the absorption spectra of B2M-CuS NPs exhibited a new peak at 250 − 300 nm that is characteristic of the antibody (Fig. 1i). The zeta potential of the NPs changed from − 7.96 mV to 1.58 mV after modification with the B2M antibody (Fig. S2). The hydrated particle size of CuS NPs was ~ 8 nm and rose to ~ 10 nm after antibody modification (Fig. S3). These results indicate the successful conjugation of the B2M antibody to CuS NPs.

We used Dox to induce ATDC5 cells into senescence. Immunofluorescence staining for p16^ink4a^ revealed that Dox-induced ATDC5 cells were intensely fluorescent (Fig. S4). Levels of p16^ink4a^ were highly correlated with age [44]; p16^ink4a^ is a senescence biomarker that induces cellular senescence through binding of CDK4 and CDK6, which prevents the downstream inhibition of the cell-cycle repressor retinoblastoma-associated protein [4, 45, 46]. We also measured senescence by the popular SA-β-gal staining method [47, 48]; the upregulation of β-galactosidase activity in lysosomes induces positive staining of senescent cells [34]. As shown in Fig. S5, ATDC5 cells stained blue after Dox-induction, which indicates SA-β-gal expression. Taken together, these results confirm the successful establishment of senescent chondrocytes.

To study the targeting specificity of B2M-CuS NPs for senescent chondrocytes, we cultured senescent ATDC5 cells with FITC-labeled B2M-CuS or FITC-labeled CuS NPs. After 4 h of incubation, green fluorescence was observed in cells cultured with FITC-labeled B2M-CuS NPs, whereas negligible fluorescence was observed in FITC-labeled CuS and control groups (Fig. 2a). We also used flow cytometry to analyze the FITC fluorescence intensity of senescent chondrocytes co-cultured with CuS and B2M-CuS NPs (Fig. 2b). As expected, FITC fluorescence intensity (Fig. 2c) was stronger in the B2M-CuS group compared to the CuS group. In contrast, targeting was suppressed by pre-treatment with free B2M antibody (Fig. S6). The targeting effect was also weak on normal chondrocytes (Fig. S7). These results indicate that NPs modified with a B2M antibody can selectively target senescent cells.

### In vitro biocompatibility and targeted elimination of senescent chondrocytes

Though Cu is toxic to mammalian cells at high doses (causing jaundice, gastrointestinal ulcers, and liver and kidney damage) [49], appropriate doses of Cu can also promote mammalian cell differentiation and tissue repair [26, 50, 51]. We investigated the biocompatibility of CuS NPs toward ATDC5 cells using a standard CCK-8 assay; neither CuS nor B2M-CuS NPs adversely affected ATDC5 cell viability after 24 h of co-culture even at NP concentrations up to 100 µg mL^− 1^ (Fig. 3a and b). Lower NP concentrations promoted the proliferation of chondrocytes, which is consistent with previous observations that Cu ions and Cu-based nanomaterials can accelerate cell proliferation by enhancing the synthesis of insulin-like growth factor 1 [52]. In hemocompatibility studies, the red color of the positive control (ddH_2_O) indicates the release of hemoglobin from ruptured erythrocytes (Fig. S8). In contrast, no red color was observed in the supernatants of CuS, B2M-CuS, and negative control (PBS) groups, indicating that the NPs are hemocompatible.

Next, we treated senescent chondrocytes with NPs to determine the specificity of killing. The CCK-8 assays (Fig. 3c) revealed that cell viability in the CuS NP group dropped to 48% of the control group, while treatment with the targeted B2M-CuS NPs reduced viability to 9% of the control. Moreover, live/dead staining further confirmed that most of the senescent chondrocytes were dead (red fluorescence) in the B2M-CuS NP group and half of the cells were dead in the CuS NP group (Fig. 3e). In contrast, cell death was negligible when normal chondrocytes were co-cultured with NPs (Fig. 3d). These results indicate that B2M-CuS NPs can specifically eliminate senescent chondrocytes without harming normal chondrocytes.

### ROS generation and cell apoptosis

To better understand the mechanism of cell death induced by NPs in senescent chondrocytes, we first measured total intracellular ROS generation via a DCFH-DA method. After entering cells, DCFH-DA converts to DCFH and forms fluorescent dichlorofluorescein when oxidized by ROS. In the control and CuS NP groups, senescent chondrocytes were weakly fluorescent (Fig. 4a and c), while cells treated with B2M-CuS NPs were intensely fluorescent, indicating substantial intracellular ROS formation. Moreover, mitochondrial ROS generation was detected using MitoROS^TM^580 as the indicator because mitochondria play a key role in cell apoptosis. MitoROS^TM^580 is a superoxide-sensitive dye that localizes to mitochondria and generates red fluorescence when oxidized by superoxide. After culturing with B2M-CuS NPs for 24 h, strong red fluorescence was observed in senescent chondrocytes (Fig. 4b). The mean fluorescence intensity was highest in the B2M-CuS NP group, indicating that B2M-CuS NPs potently induce mitochondrial ROS formation in senescent cells (Fig. 4d). ROS were also detected in senescent chondrocytes cultured with bald CuS NPs, which still have peroxidase-like activity but lack a cell-targeting domain. In comparison, cells of the control group were weakly fluorescent. The levels of intracellular ROS and mitochondrial ROS in normal chondrocytes were negligible (Fig. S9). Mitochondrial ROS formation leads to a decline in mitochondrial function, which in turn increases ROS production, thus forming a feedback loop that promotes apoptosis. Notably, B2M-CuS NPs are safe for normal chondrocytes because they are targeted specifically to senescent chondrocytes, which have greater ROS sensitivity than normal cells [53, 54].

We further studied the expression of representative apoptosis-related genes, including *Bcl-2*, *Bax*, *caspase-9*, and *caspase-3*. The anti-apoptotic protein Bcl-2 resides in the outer mitochondrial membrane and inhibits cytochrome C release, while pro-apoptotic protein Bax resides in the cytosol and translocates to the mitochondria during apoptosis. Compared with controls, treatment of senescent chondrocytes with B2M-CuS NPs resulted in reduced expression of anti-apoptotic *Bcl-2* and increased expression of pro-apoptotic Bax (Fig. 4e and f). An increase in the Bax/Bcl-2 ratio (Fig. 4g) promotes the release of cytochrome c via mitochondrial permeabilization, thus activating the initiator caspase-9 through the intrinsic pathway [55, 56]. Caspase-9 then cleaves and activates the effector caspase-3, leading to the cleavage of specific intracellular proteins and cell death [57, 58]. Among all treatment groups, the levels of *caspase-9* and *caspase-3* gene expression were highest in senescent chondrocytes treated with B2M-CuS NPs (Fig. 4h and i), and flow cytometry analysis (Fig. 4j and Fig. S9) confirmed this finding. 7-AAD staining also revealed that cell death is highest in the B2M-CuS NP treatment group (Fig. 4k and Fig. S10). B2M is a well-recognized membrane marker of senescence that can be used to selectively target senescent cells, and the modification of CuS NPs with the B2M antibody enables their cellular uptake by senescent cells. We found that this targeting was suppressed by pre-treatment of senescent cells with free B2M antibody. Taken together, these results indicate that B2M-CuS NPs can efficiently eliminate senescent cells through the conversion of H_2_O_2_ into toxic ROS that induce apoptosis.

### In vitro induction of chondrogenesis by B2M-CuS NPs

Following the elimination of senescent cells, the NPs are expected to promote the chondrogenesis of normal chondrocytes to facilitate cartilage repair. To evaluate this chondrogenesis, ATDC5 cells were cultured in chondrogenic medium supplemented with CuS NPs, B2M-CuS NPs, or PBS. The synthesis and remodeling of Acan occurs at early stages of cartilage formation and remains low during the middle and late stages [59–61], while Col-2 expression is consistently high throughout the early and late stages of cartilage formation [62]. We found that *Acan* gene expression was significantly higher in chondrocytes co-cultured for 7 days with B2M-CuS and CuS NPs, as compared to the control group (Fig. 5a), though by day 14 this difference was no longer statistically significant (Fig. 5b). Moreover, *Col-2* expression was also up-regulated in NP groups relative to the control group (Fig. 5c, d), and was highest in cells treated with B2M-CuS NPs.

The deposition of GAG was detected by alcian blue staining. GAG is a linear heteropolysaccharide that accelerates the proliferation and differentiation of chondrocytes to promote articular cartilage regeneration [63]. Cells cultured with B2M-CuS and CuS NPs stained more deeply than cells in the control group (Fig. 5e), indicating that the NPs had increased GAG formation. Moreover, ATDC5 cells were chondrogenically induced into pellets and cultured with the NPs for 21 days. Cell pellets cultured with B2M-CuS and CuS NPs stained more deeply for Col-2 than the control group (Fig. 5f), demonstrating that the NPs promote Col-2 expression in chondrocytes. These results are consistent with our previous findings that Cu ions and Cu-based nanomaterials promote chondrogenesis [26–28, 38].

### In vivo therapeutic effect of B2M-CuS NPs in OA mice

To investigate the therapeutic effect of B2M-CuS NPs on OA, we established a DMM mouse model and delivered NPs into knee joints via intra-articular injection on a q14dx2 course (every 2 weeks for 4 weeks; Fig. 6a). Eight weeks after surgery, safranin O/fast green staining (Fig. 6c) of control PBS-treated DMM samples revealed the severe destruction of articular cartilage at the tibial plateau (highlighted by red arrows) and exposure of the subchondral bone, thus validating the OA model. Intra-articular injection of NPs (especially B2M-CuS NPs) was protective against articular cartilage damage. The severity of cartilage destruction was further analyzed by OARSI score (Fig. 6b); OARSI scores in the PBS groups were greater than those of the sham group at post-surgical week 8. In contrast, OARSI scores were lowest in mice treated with B2M-CuS NPs.

Cartilage expression of both Col-2 (Figs. 6d and 7a) and matrix degradation marker MMP-13 (Figs. 6e and 7b) are similar between B2M-CuS NP and sham groups, but in control (PBS) treated OA mice, Col-2 expression is reduced while MMP-13 expression is increased. Hence, B2M-CuS NPs are the most effective OA treatment in this study.

Furthermore, the expression of p16^ink4a^ and HMGB-1 in joint cartilage was investigated by immunofluorescence staining to confirm the in vivo elimination of senescent chondrocytes by B2M-CuS NPs. p16^ink4a^ is a cyclin-dependent kinase inhibitor that activates retinoblastoma-associated protein and induces cellular senescence [64], while the loss of nuclear HMGB-1 is considered a senescence biomarker [16, 44]. Intra-articular injection of B2M-CuS NPs reduced p16^ink4a^ expression (Figs. 6f and 7c) and increased HMGB-1 expression (Figs. 6g and 7d) in OA mouse cartilage, while the opposite trend was observed in the control PBS group. Thus, B2M-CuS NPs effectively eliminate senescent chondrocytes in vivo. Additionally, H&E staining (Fig. S12) of the main organs indicates that the NP treatments generated no adverse effects. We also analyzed the biodistribution of B2M-CuS NPs in mice 24 h after intra-articular injection and found that Cu^2+^ content was higher in the knee joint, liver, and kidney (Fig. S13).

## Conclusion

In this study, we synthesized ultrasmall CuS NPs with peroxidase-like properties and then functionalized them with B2M antibodies. These B2M-CuS NPs selectively target and eliminate senescent chondrocytes by inducing apoptosis via toxic ROS formation. After clearing senescent chondrocytes, B2M-CuS NPs promote the chondrogenesis of normal chondrocytes and the synthesis of GAG and Col-2. In DMM-induced OA mice, we found that intra-articular injection of B2M-CuS NPs effectively eliminates senescent chondrocytes and enhances cartilage regeneration. Our study effectively treated OA via a synergistic strategy of anti-senescence and pro-chondrogenesis that may inform the treatment of OA and other degenerative joint diseases.

### Electronic supplementary material

Below is the link to the electronic supplementary material.

Supplementary Material 1

## Data Availability

All data generated or analyzed during this study are included in this published article and the Supporting Information.
