# Supplementary material for: Conversion of senescent cartilage into a pro-chondrogenic microenvironment with antibody-functionalized copper sulfate nanoparticles for efficient osteoarthritis therapy
Source: J Nanobiotechnology. 2023 Aug 8;21:258. doi: 10.1186/s12951-023-02036-5 (PMC10408088; doi:10.1186/s12951-023-02036-5)
Supplement: Supplementary file 1 — Supplementary Material 1 [file 12951_2023_2036_MOESM1_ESM.docx]

Supplementary Materials

**Conversion of senescent cartilage into a pro-chondrogenic microenvironment with antibody-functionalized copper sulfate nanoparticles for efficient osteoarthritis therapy**

Xianming Wang^a,b,c#^, Yu Cai^d#^, Cuixi Wu^e#^, Jiamin Liang^f^, Kangning Tang^c^, Zefeng Lin^c^, Lingling Chen^c^, Yao Lu^e*^, Qing Wang^a,b*^

^a^Department of Orthopedic Surgery, The Second Affiliated Hospital, Guangzhou Medical University, Guangzhou, Guangdong, China.

^b^The First School of Clinical Medicine, Southern Medical University, Guangzhou, Guangdong, China

^c^Department of Orthopedics, General Hospital of Southern Theater Command of PLA, Guangzhou, Guangdong, China

^d^Precision Medicine in Oncology (PrMiO), Department of Pathology, Erasmus MC Cancer Institute, Erasmus MC, Rotterdam, The Netherlands

^e^Department of Joint and Orthopedics, Orthopedic Center, Zhujiang Hospital, Southern Medical University, Guangzhou, Guangdong, China

^f^Department of Endocrinology, Zhujiang Hospital, Southern Medical University, Guangzhou, Guangdong, China

# These authors contributed equally to this work.

* Corresponding author: E-mail: oayul@smu.edu.cn (Y. Lu); wq748911@163.com (Q. Wang).


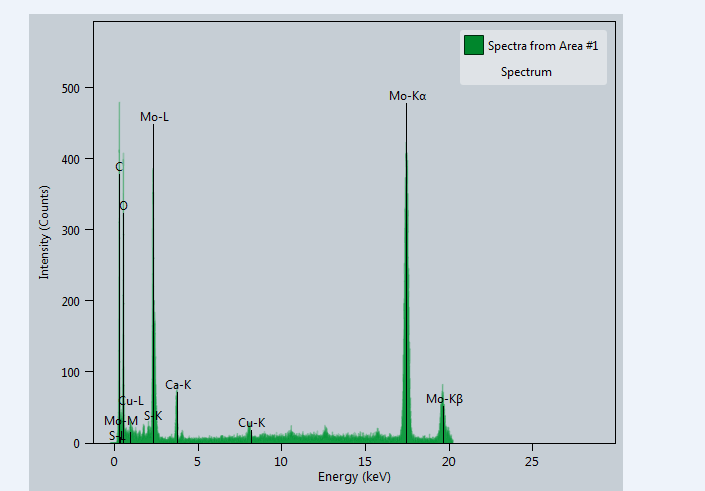


**Figure S1.** EDS spectra of CuS NPs. Noted that the presence of Mo is due to the use of molybdenum TEM grid.


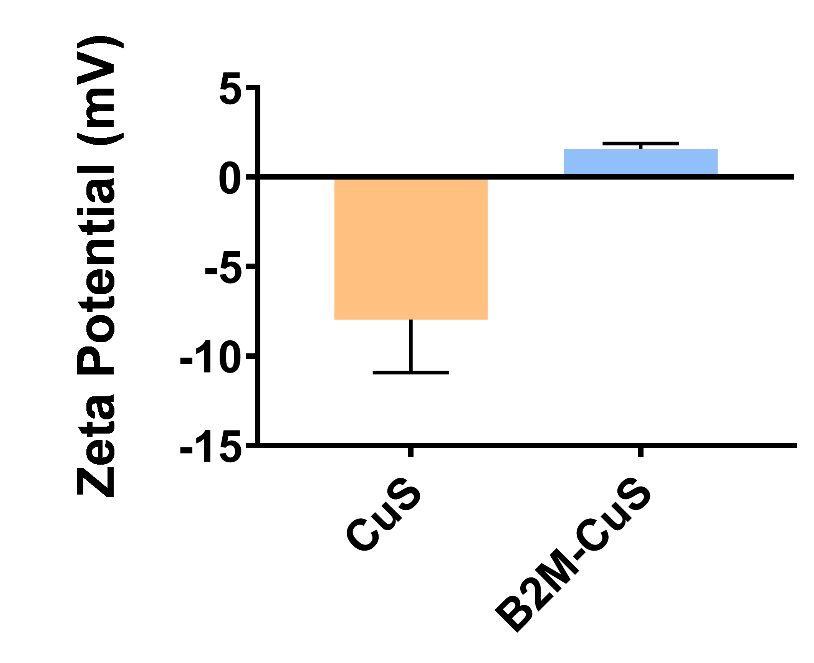


**Figure S2.** Zeta potential of NPs.


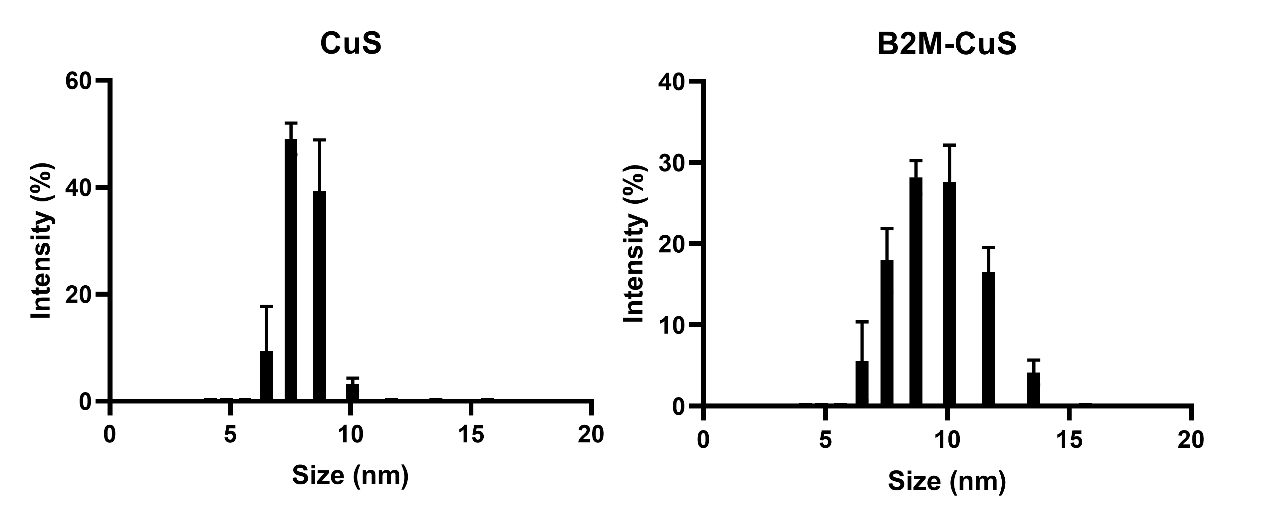


**Figure S3.** Hydrated particle size of NPs.


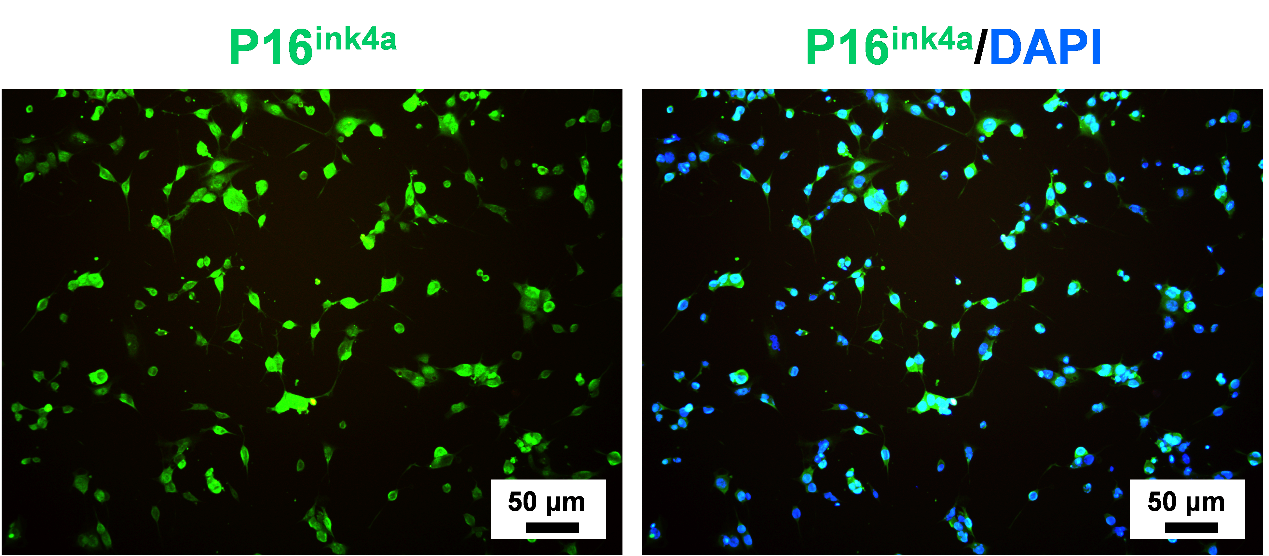


**Figure S4.** Immunofluorescence staining of p16^ink4a^ in senescent cells.


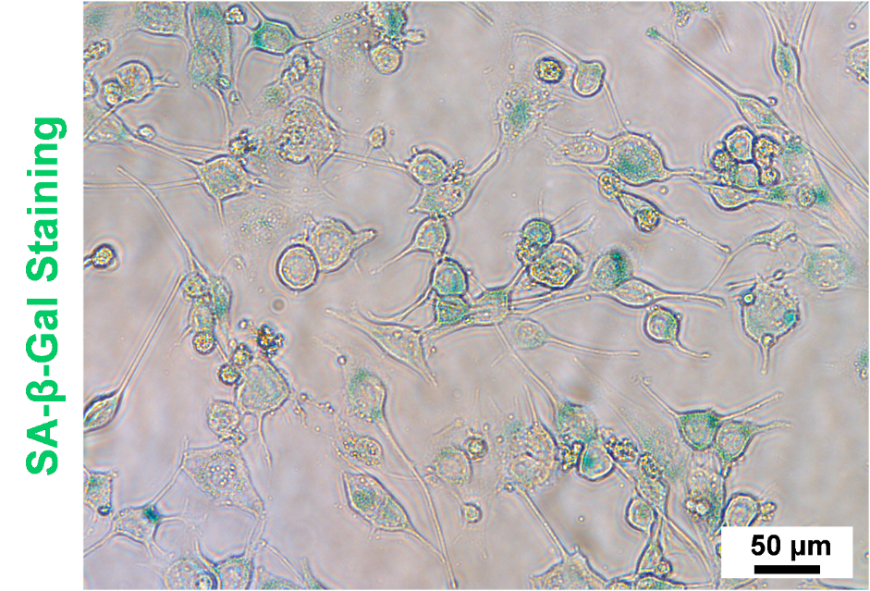


**Figure S5.** SA-ꞵ-GAL staining of senescent cells.


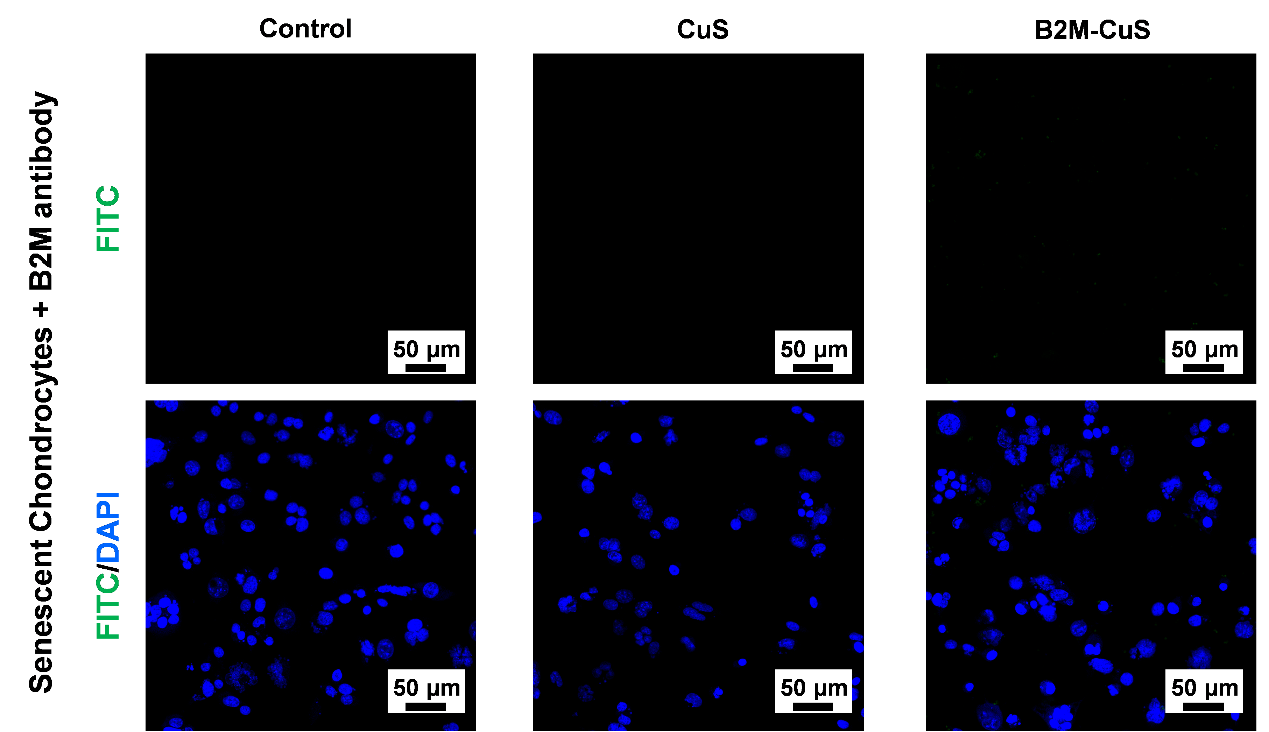


**Figure S6.** Targeting effect of B2M-CuS toward senescent cells in the presence of free B2M antibody.


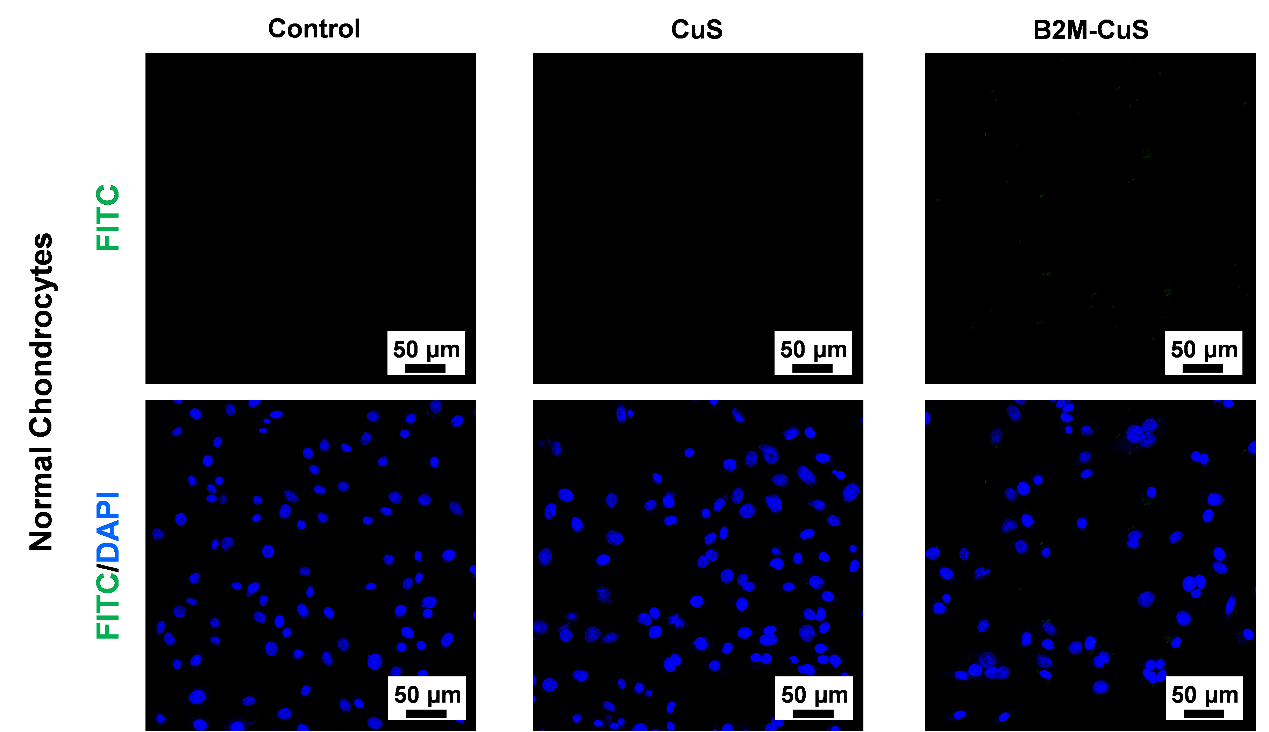


**Figure S7.** Targeting effect of NPs toward normal chondrocytes.


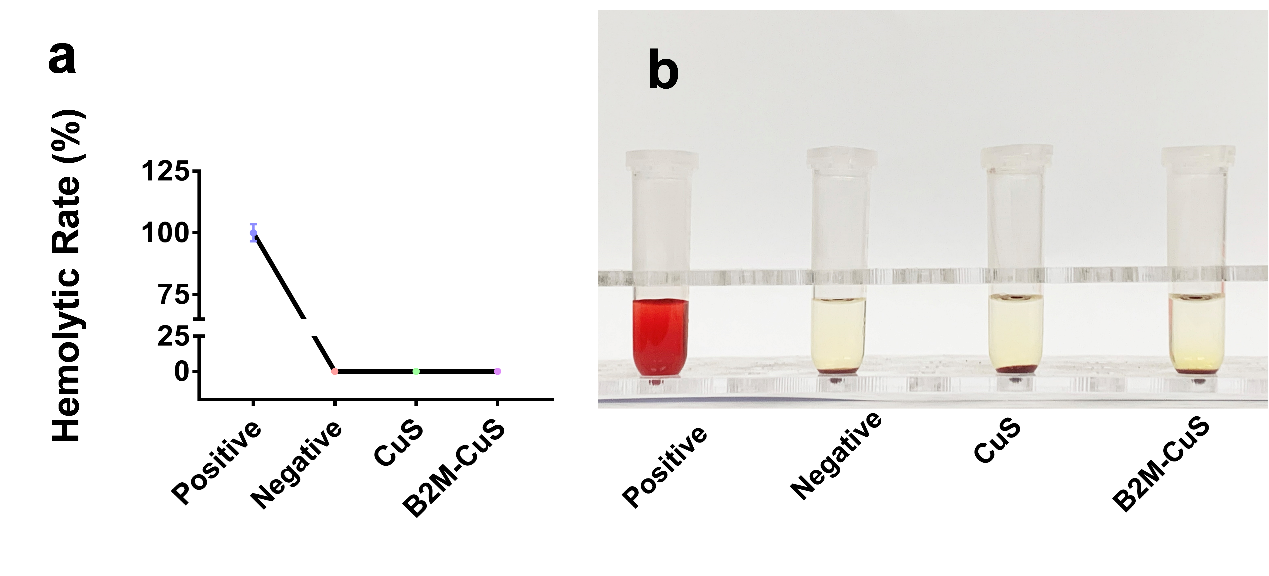


**Figure S8.** Hemolysis study of NPs. (a) Hemolytic rate of CuS and B2M-CuS NPs. (b) General image of blood with different treatments.


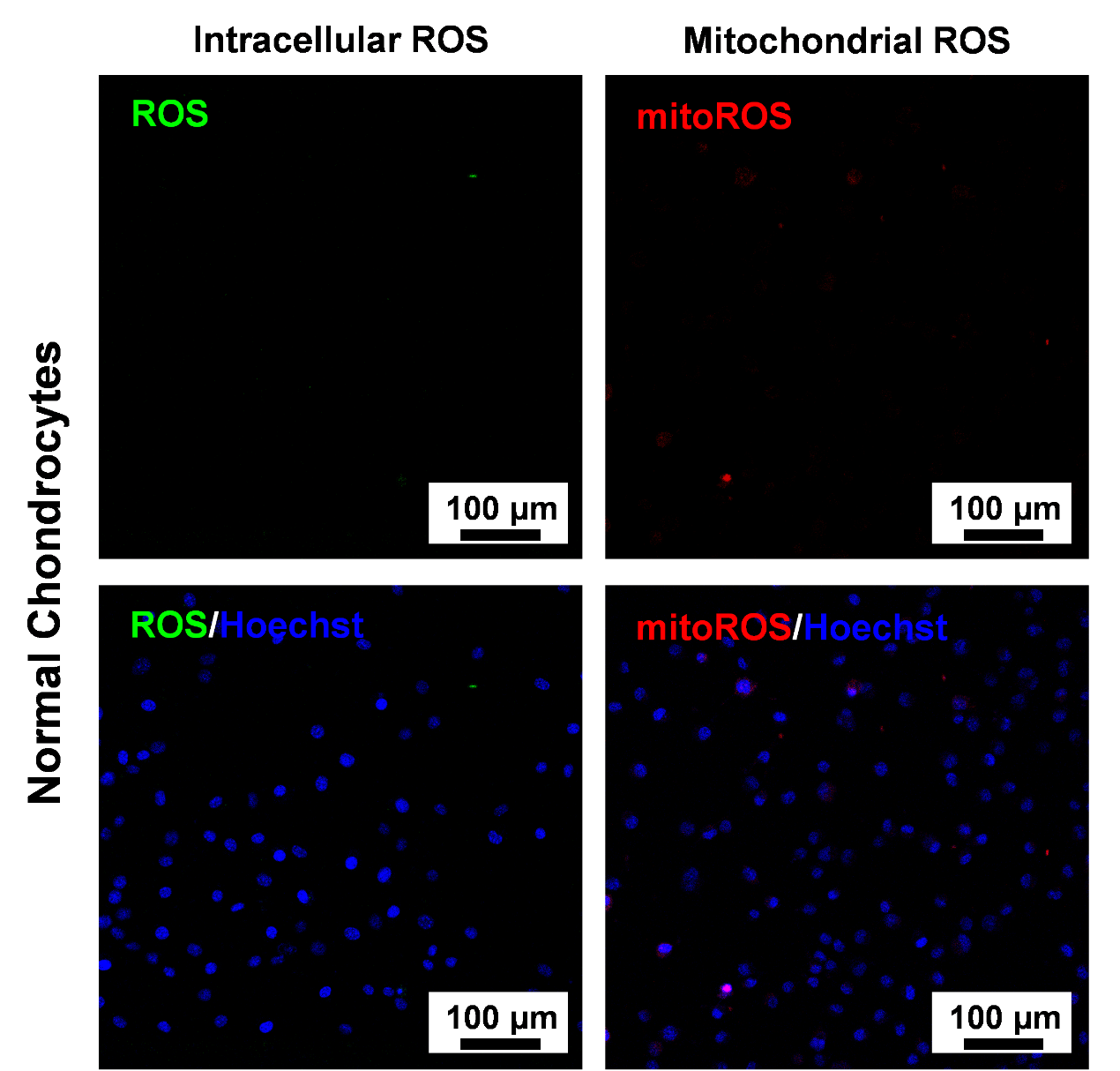


**Figure S9.** Intracellular ROS and mitochondrial ROS levels in normal chondrocytes.


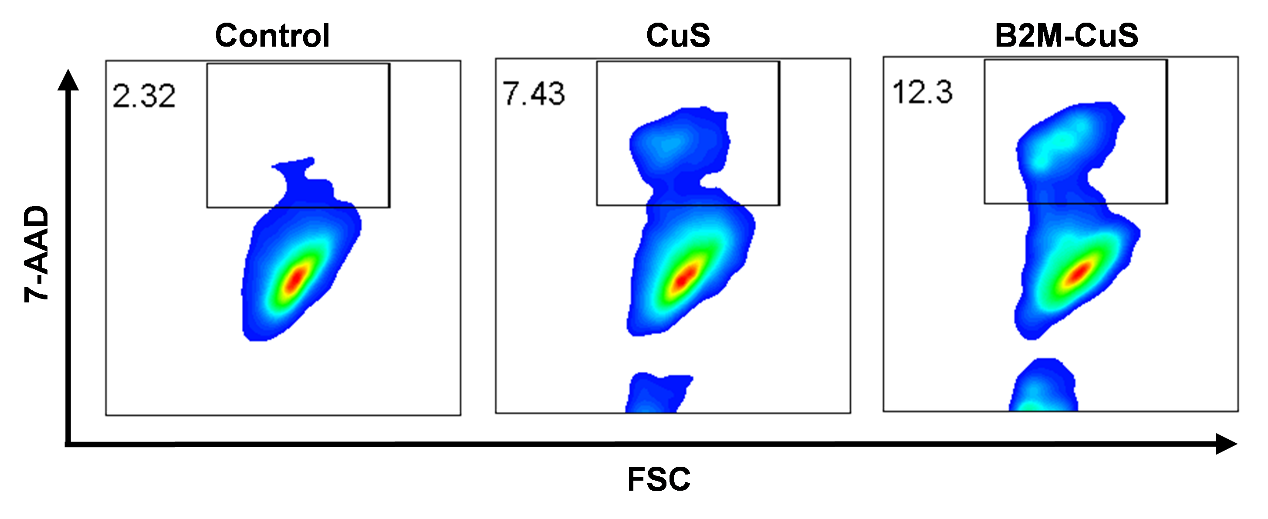


**Figure S10.** Representative flow cytometry images of 7-AAD staining.


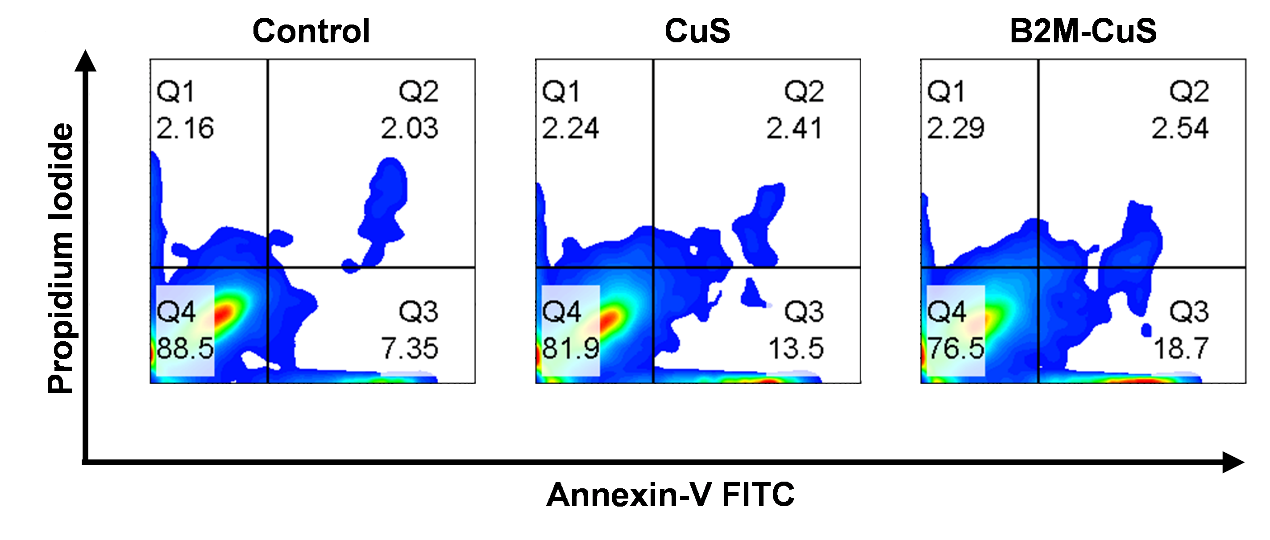
 **Figure S11.** Representative flow cytometry analysis of apoptosis in senescent chondrocytes following treatment.


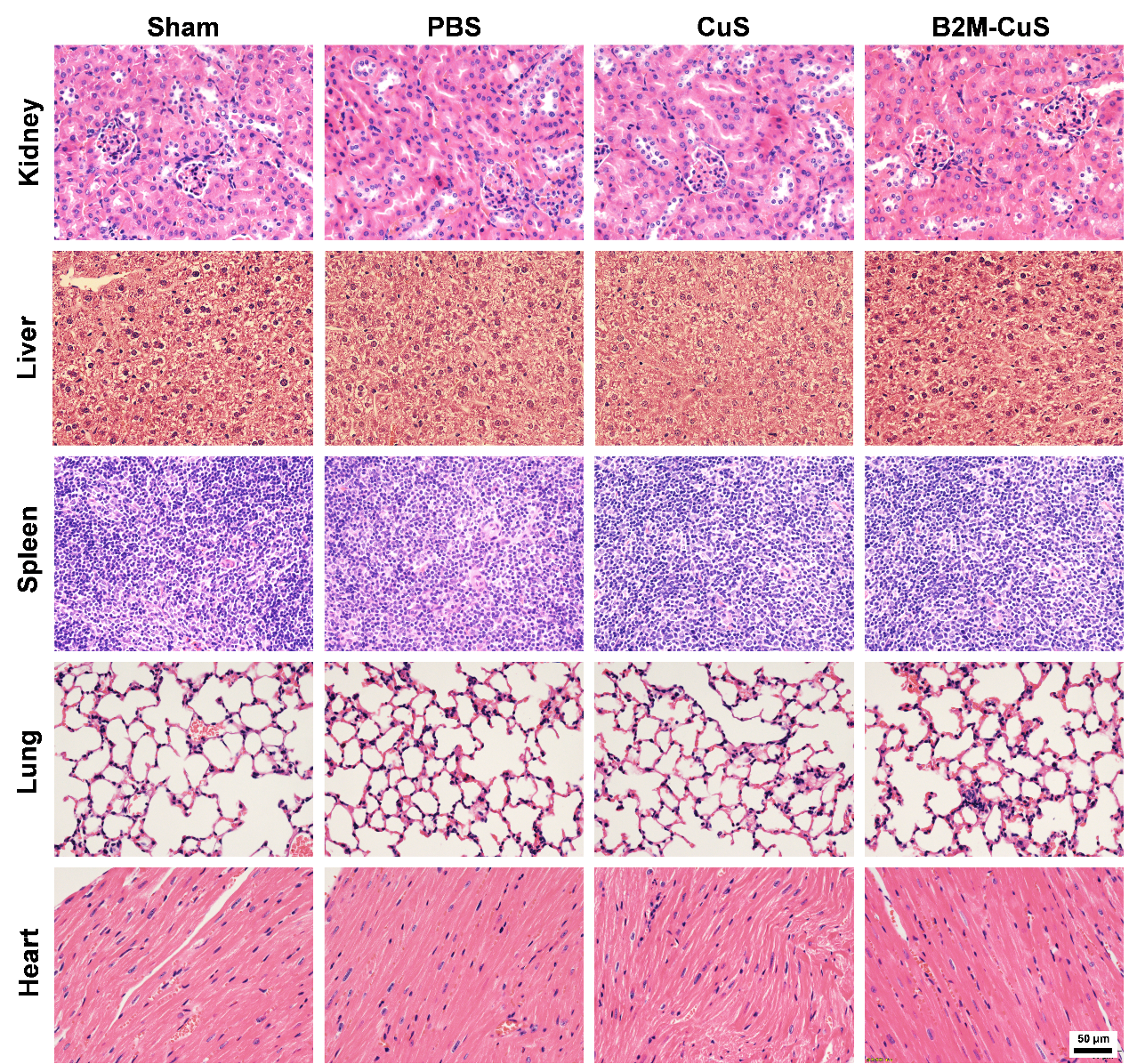


**Figure S12.** H&E staining of the main organs of mice after different treatments.


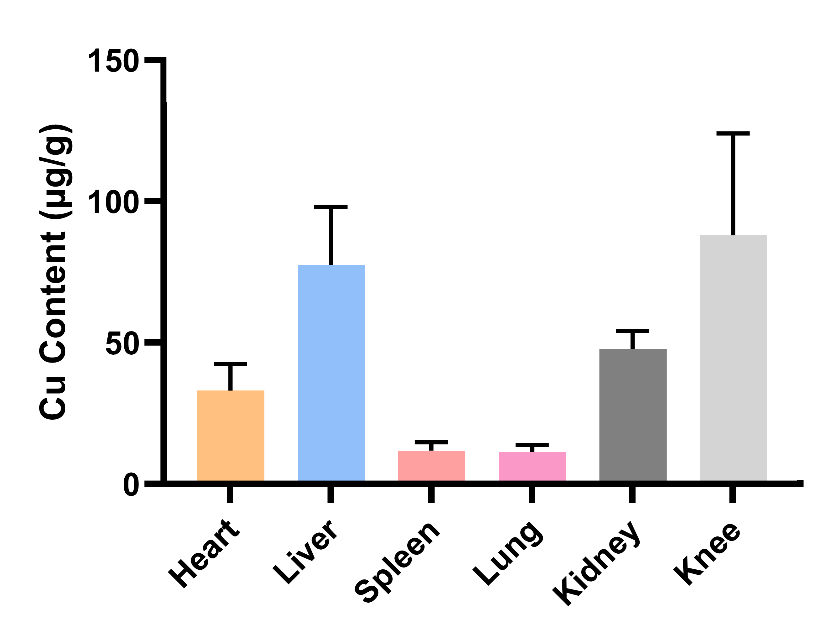


**Figure S13.** Cu^2+^ content in the main organs of mice 24 h after intra-articular injection of B2M-CuS NPs.

**Table S1.** Primers used for qRT-PCR

| Genes | Sequence (5’-3’) |
| --- | --- |
| GAPDH | Forward: GAGGTATCCTGACCCTGAAGTA |
|  | Reverse: CACACGCAGCTCATTGTAGA |
| Caspase 3 | Forward: AGTGGGACTGATGAGGAGAT |
|  | Reverse: GAGACATCTCCTTCCCGTTTAC |
| Caspase 9 | Forward: GGCTTCATTTGGTTGGTTGG |
|  | Reverse: GGCCAGAACTTGGGAATAAGA |
| Bax | Forward: GTGGTTGCCCTCTTCTACTTT |
|  | Reverse: CAGCCCATGATGGTTCTGAT |
| Bcl-2 | Forward: GAGCAGGTGCCTACAAGAAA |
|  | Reverse: CTTTGTCCTCTGACTGGGTATG |
| Aggrecan | Forward: ATTTCCACACGCTACACCCTG |
|  | Reverse: TGGATGGGGTATCTGACTGTC |
| Col-2 | Forward：AGATGGTCCCAAAGGTGTTC |
|  | Reverse: CATCTCCAGGTTCTCCTTTCTC |
